# The Formula for Better Health

**DOI:** 10.3201/eid3208.260497

**Published:** 2026-08

**Authors:** David M. Brett-Major

**Affiliations:** University of Nebraska Medical Center, Omaha, Nebraska, USA

**Keywords:** Public health, messaging, pandemics, tuberculosis, Ebola, emerging diseases

In The Formula for Better Health, Dr. Tom Frieden relates a series of personal and historical experiences at pivotal local and global moments in public health to frame useful approaches to improve outcomes in the future (Figure). He introduces those moments with reverence for his predecessors and colleagues, narrating personal experiences that build familiarity with existential health threats that are felt locally. In doing so, he reinforces a stepwise argument for how to attack public health problems that affect millions if not billions of persons, such as hypertension and heart disease, consequences of tobacco use, lead poisoning, tuberculosis, HIV, emerging infectious diseases such Ebola disease and COVID-19, and poverty challenges to wellness and health access. I felt 2 quotes from the middle of the book exemplified his thoughts on public health action and messaging: “Simplicity is the ultimate sophistication,” by Leonardo Da Vinci; and “…the fierce urgency of now,” by the Reverend Dr. Martin Luther King, Jr.

**Figure:**
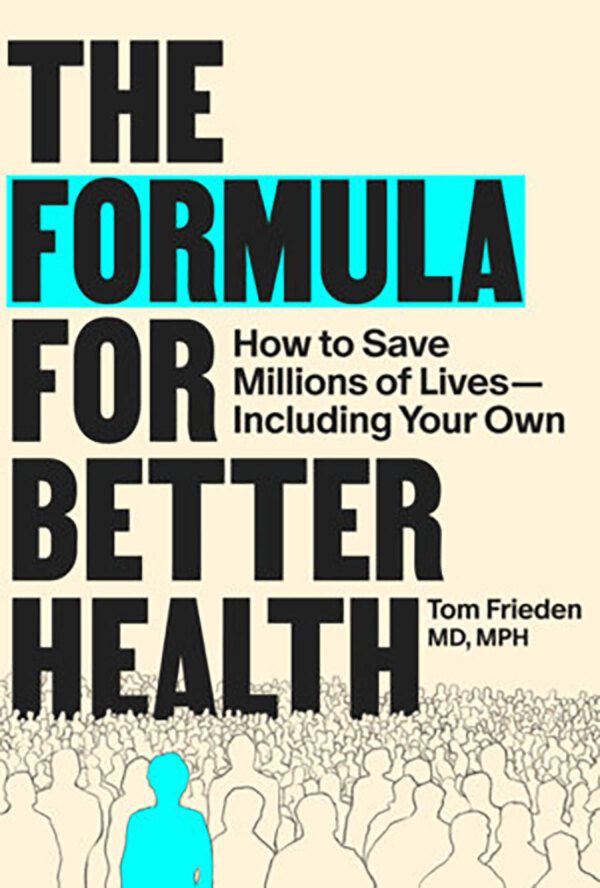
The Formula for Better Health.

Structurally, Dr. Frieden organized his reflections in 4 parts: see, believe, create, and the formula in practice. His underlying premise is that to successfully address a health challenge we must first detect it and understand its effect through the appropriate analysis of quality data (see); we then should arrive at a consensus on the need for action by operating in an environment of trust and shared interests (believe); and finally we can develop and apply a solution that directly attacks the specific problem (create). In each part, readers are introduced and reintroduced to different aspects of his work as he elucidates this framework. This approach results in an iterative reinforcement of each component. For instance, in part 3, create, he frequently references the see and believe aspects of specific experiences mentioned earlier in the book.

The narrative cannot help but transmit Dr. Frieden’s New Yorker roots, both in a professional context, working in tuberculosis management, and culturally. I paraphrase Mark Twain’s remarks on being in New York City when I say The Formula for Better Health, too, is a busy place. The book is packed with experiential narratives and other information. Nonetheless, it is carefully annotated and has useful summary tables and appendices. In conveying the most intense experiences, such as around Ebola disease in West Africa and the United States, or tuberculosis or tobacco use in New York, Dr. Frieden is anchored in organizational loyalty and a singular focus on project objectives, although the directions he takes from these experiences were constructive.

His approach is consistent with a lifetime of messaging and the work of his effective nonprofit organization, Resolve to Save Lives. Dr. Frieden makes his case both reasonably and emphatically, successfully writing content that is digestible by members of the public, public health and healthcare professionals, and policy makers. The lay reader of The Formula for Better Health will get a compelling, broad familiarization to pivotal issues and concepts in public health, relevant to both communicable and noncommunicable diseases. Technically experienced readers will gain perspective on the views and approach of an important champion for our nation’s health who is committed to a strong Centers for Disease Control and Prevention. They also will gain advice for how to address complicated aspects of managing health risks through both public messaging and program design.

